# Supplementary material for: Extracorporeal Shock Wave Therapy Combined with Platelet-Rich Plasma during Preventive and Therapeutic Stages of Intrauterine Adhesion in a Rat Model
Source: Biomedicines. 2022 Feb 17;10(2):476. doi: 10.3390/biomedicines10020476 (PMC8962268; doi:10.3390/biomedicines10020476)
Supplement: Supplementary file 1 [file biomedicines-10-00476-s001.zip › biomedicines-1595233-supplementary.pdf]

**Supplementary Data**

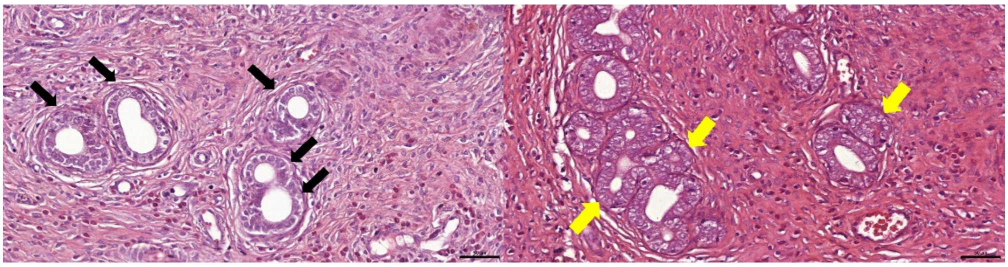

H&E-stained sections of SD rat uterine tissue after curettage. Uterus of control group showing normal uterine architecture, we counted the number of glands that were following these principles. Bar = 50 μm. The uterus of the control group showing normal uterine architecture with an intact endothelium, presenting normal different uterine layers (Black Arrows); uterine architecture with uncomplete endothelium (Yellow Arrows) H&E; hematoxylin and eosin;
SD, Sprague-Dawley.
